# Synthesis and assessment of ionic liquid derived from benzalkonium chloride as corrosion inhibitor for carbon steel

**DOI:** 10.1038/s41598-025-14549-0

**Published:** 2026-02-09

**Authors:** Ashraf M. Ashmawy, Reda Abdel-Hameed, Odeh A. O. Alshammari, Maher I. Nessim, Modather F. Hussein, Abdalrahman G. Al-Gamal

**Affiliations:** 1 Chemistry Department, Faculty of Science (Boys), Al-Azhar University, Cairo, 11884 Egypt; 2 Department of Chemistry, College of Science, University of Ha’il, Hail, 81442 Saudi Arabia; 3 Egyptian Petroleum Research Institute (EPRI), Nasr City 11727, Cairo, Egypt; 4 Chemistry Department, Faculty of Science, Al-Azhar University, Asyut Branch, Assiut, 71524 Egypt

**Keywords:** ILs, EIS, EFM, Benzalkonium chloride, HOMO, LUMO, Chemistry, Green chemistry, Ionic liquids

## Abstract

Corrosion poses a significant challenge in the materials industry, especially when carbon steel (C-steel) is subjected to highly corrosive environments such as 1 M HCl. In this study, four ionic liquids (ILs)-1-benzyl-1-methylpiperidin-1-ium chloride (IL_I_), 1-benzyl-2-methylpyridin-1-ium chloride (IL_II_), 1-benzyl-3-methylpyridin-1-ium chloride (IL_III_), and 1-benzyl-4-methylpyridin-1-ium chloride (IL_IV_) were synthesized and characterized using spectroscopic techniques, including FT-IR and ^1^HNMR. Electrochemical analyses such as potentiodynamic polarization (PP), electrochemical impedance spectroscopy (EIS), and electrochemical frequency modulation (EFM) demonstrated excellent protective performance for C-steel in 1 M HCl, reaching a maximum inhibition of 97% at an optimum concentration of 100 ppm. Insights from polarization and impedance analyses confirmed that these compounds act as mixed-type inhibitors by adhering to the steel surface. The adsorption behavior followed the Langmuir isotherm model, with ΔG^0^ ads values suggesting a combination of physisorption and chemisorption. Quantum chemical assessments validated the experimental results, indicating that IL_II_–IL_IV_, due to their two aromatic rings, exhibited superior surface interaction and inhibition efficiency because of their reduced HOMO-LUMO energy gaps. These findings affirm the strong inhibitory potential of the tested ILs in acidic media.

**Supplementary Information:**

The online version contains supplementary material available at 10.1038/s41598-025-14549-0.

## Introduction

Ionic liquid (IL) compounds are widely utilized as anticorrosive agents due to several advantages, including high efficiency, environmental compatibility, and cost-effectiveness^1–4^. Owing to their inherent hydrophilic-lipophilic properties, ILs can readily form micelles by reducing surface tension, thereby optimizing the balance between adhesion and cohesion forces. This leads to improved adhesiveness and enhanced adsorption capacity^5^. Their distinctive characteristics make ILs an excellent alternative to replace hazardous materials in industrial applications, and they are frequently employed as solvents in numerous chemical processes^6^. Recently, researchers have shown increasing interest in exploring the behavior of ILs as corrosion inhibitors (CIs)^7–11^.

Deyab and Mohsen investigated the corrosion inhibition performance of a phosphate-based IL, specifically Tributylmethylphosphonium Bis(trifluoromethanesulfonyl)imide (PBIL), for steel structures exposed to brine water containing H₂S and CO₂ at a pH = 2. Their findings demonstrated that PBIL achieved an inhibition efficiency of nearly 92% at a concentration of 100 ppm^12^. In another study, Pisanenko, Klimko et al. synthesized three IL derivatives—3-(N-arylcarboxamido)-N-benzyl pyridinium chlorides—where the aryl groups were phenol (I), 1-naphthyl (II), and 2-naphthyl (III). These compounds were evaluated for their anticorrosion performance against 08 kp steel in 3 M hydrochloric acid within the temperature interval of 20–80 °C. The results indicated that ILs II and III exhibited significant anticorrosion activity^13^.

To accurately describe the behavior of these molecules, factors such as the aromatic structure, along with accessible π-electrons or free electron pairs outside the coordination sphere, play a crucial role in determining their adsorption on metal surfaces. Once adsorbed, these molecules obstruct active sites, thereby mitigating the corrosion rate^14–16^.

Oilfield corrosion creates numerous operational challenges, triggering leaks in critical infrastructure, including tanks, casings, tubing, and pipelines, that adversely affect both production processes and maintenance procedures^17,18^. Such issues often lead to repeated partial or complete shutdowns, accounting for over 20% of the maintenance budget, resulting in substantial financial losses^19^. “Despite their extensive use in industrial processes such as pickling, cleaning, and descaling, acids accelerate the degradation of steel-based equipment, especially C-steel. Therefore, this study is dedicated to synthesizing four ILsand assessing their corrosion inhibition performance for C-steel in 1 M HCl.”

## Experimental

### Materials

1. The precursor chemicals required for the synthesis four target ILs were procured. Benzylwasride (99%), 1-methylpiperidine (98%), 2-picoline (98%), 3-picoline (98%), and 4-picoline (97%) were obtained from Alfa Aesar, while acetonitrile and diethyl ether were sourced from Merck. All chemicals and reagents were utilized as received without undergoing further purification.
2. Chemical Composition of the Tested C-steel.

The C-steel employed in this study had the following chemical composition (wt.%): Fe (balance), Mn 0.853%, C 0.093%, Al 0.032%, Cr 0.025%, Ni 0.013%, P 0.014%, Si 0.011%, and Cu 0.012%.”

3. Corrosion Medium.

A 1 M HCl solution was prepared as the corrosive medium by diluting 37% HCl (AR grade) with distilled water. The concentration range of the synthesized ILs compound used in the study varied between 20 and 100 ppm.

### Synthesis of ionic liquids (IL_I_-IL_IV_)

**Fig. 1:**
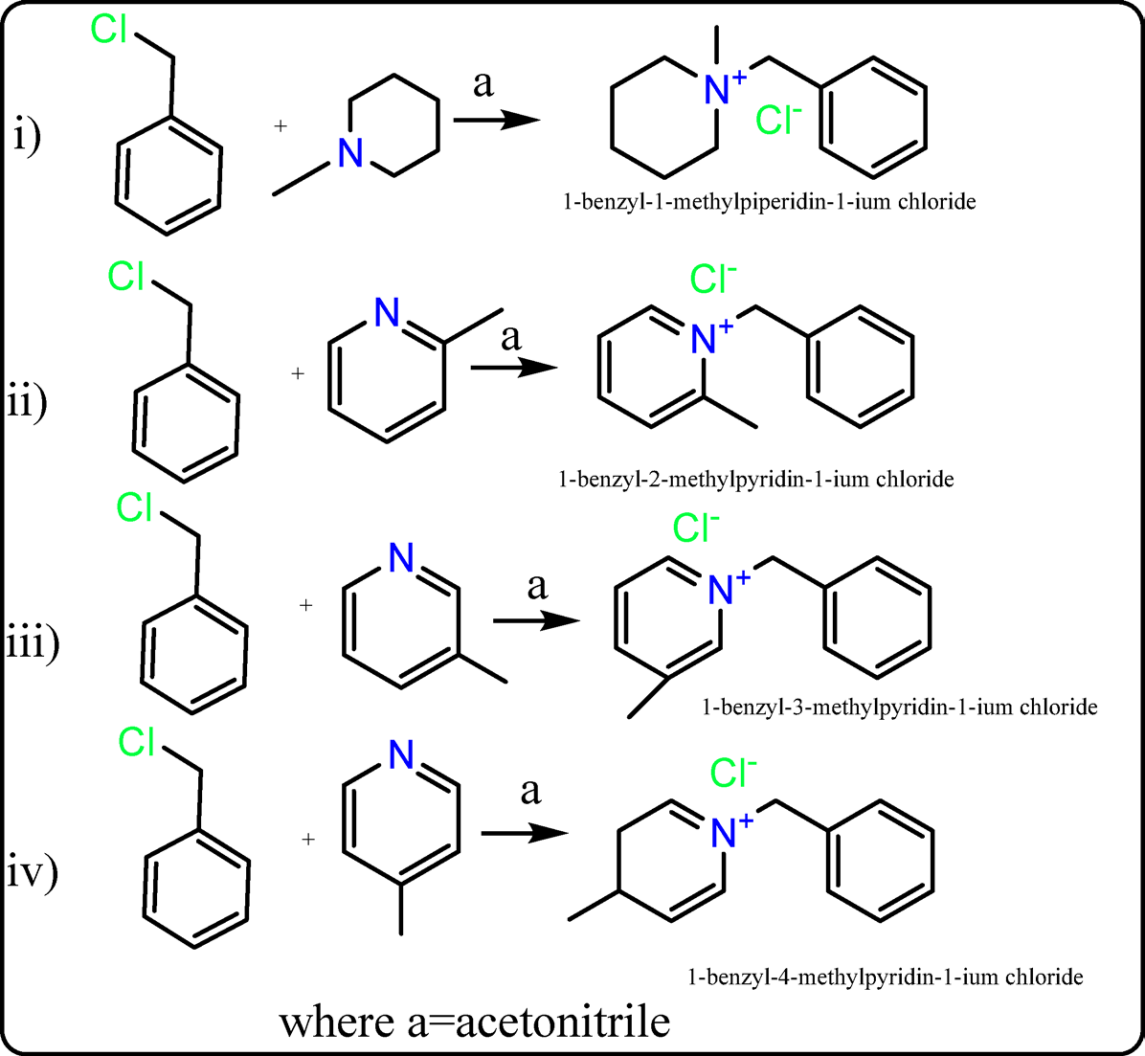
Preparation of ionic liquids IL_I_-IL_IV_.

The four compounds (IL_I_–IL_IV_) have been synthesized previously using various methods^20–22^. In this study, they were prepared following the procedure described in the reference^23^, as illustrated in Fig. 1. A solution of benzyl chloride (0.05 mol) in acetonitrile was mixed with equimolar amounts (0.05 mol) of 1-methylpiperidine, 2-picoline, 3-picoline, and 4-picoline. The reaction mixture was subjected to reflux at 80 °C for 6 h. After completion, the resulting solution was evaporated under vacuum, and the obtained products (IL_I_–IL_IV_) were thoroughly washed multiple times with diethyl ether^20–22^. A flowchart of the research method in Fig. S1.

### Characterization of ionic liquid

To confirm the molecular structure of the synthesized ILs, elemental composition was determined using a Perkin Elmer 240 C elemental analyzer. The infrared (IR) spectra were measured on a Nicolet iS10 spectrometer within the range of 4000 to 400 cm^− 1^, applying a resolution of 4 cm^− 1^ and a scanning speed of 32 cm/min. Additionally, proton nuclear magnetic resonance (^1^H NMR) spectra were recorded on a BRUKER 400.19 MHz spectrometer, equipped with a 5-mm broadband inverse Z-gradient probe, utilizing DMSO-d_6_ as the solvent.”

### Electrochemical measurement

Steel (C-steel) with a surface area of 1 cm^2^ served as the working electrode. The reference electrode was a saturated calomel electrode (SCE), and a platinum wire acted as the counter electrode. Various electrochemical techniques, including impedance spectroscopy (EIS), polarization measurements, and electrochemical frequency modulation (EFM), were sequentially performed in the same experimental setup. The EIS measurements were carried out at open circuit potential (OCP) with a 10 mV alternating voltage applied, covering a frequency range from 100 kHz to 20 mHz at 25 °C. For EFM, a 10 mV AC amplitude was applied at 2 and 5 Hz frequencies. Polarization curves for anodic and cathodic surface interactions were also measured with a voltage sweep of ± 1.5 V/SCE relative to OCP at a scan rate of 5 mV/s. All data from the experiments were processed using the Echem Analyst software, with the measurements being carried out using a Gamry 3000 Potentiostat/Galvanostat/ZRA^3,24–27^.

### Quantum chemical calculations

The present investigation focused on molecules that were designed and optimized geometrically through the DMol^3^ calculation model in Materials Studio v7.0. The geometry optimization process employed high-quality settings, utilizing the Local Density Approximation (LDA) functional and the DNP basis set, with all electrons treated for the core. Key quantum chemical parameters were evaluated, including the energies of the highest occupied molecular orbital (E_HOMO_) and the lowest unoccupied molecular orbital (E_LUMO_), as well as the energy gap (ΔE = E_LUMO_ - E_HOMO_)^28,29^. The ionization potential (I) was determined from -E_HOMO_ and the electron affinity (A) was calculated as -E_LUMO_. Electronegativity (χ) was derived as the average of I and A, while global hardness (η) was computed as their difference divided by 2. Softness (σ) was calculated as the reciprocal of η, and the electrophilicity index (ω) was given by ω = µ^2^/2η, where µ = (I - A)/2 denotes the chemical potential. For reference, the electronegativity and hardness of iron (χFe ≈ 7 eV and ηFe = 0) were assumed. Additionally, the Fukui indices for nucleophilic (f^+^) and electrophilic (f^−^) local reactivity were calculated using the DMol^3^ method^30–35^.

## Results and discussion

### Characterization of IL_I_-IL_IV_ structures

#### Elemental analysis

The data shown in Table 1 demonstrate that the calculated percentages for each element align with the theoretical predictions.

**Table 1:** Elemental analysis results of ionic liquid (IL_I_-IL_IV_).

Cpd.	C%		H%		*N*%		Cl%	
	Calc.	Obs.	Calc.	Obs.	Calc.	Obs.	Calc.	Obs.
IL_I_	69.16	68.96	8.93	8.04	6.20	6.90	15.70	16.01
IL_II_	71.07	71.92	6.42	6.95	6.38	6.04	16.13	16.91
IL_III_	71.07	72.01	6.42	6.81	6.38	5.87	16.13	15.89
IL_IV_	71.07	70.92	6.42	5.82	6.38	5.95	16.13	17.01

#### FT-IR spectroscopy

**Fig. 2:**
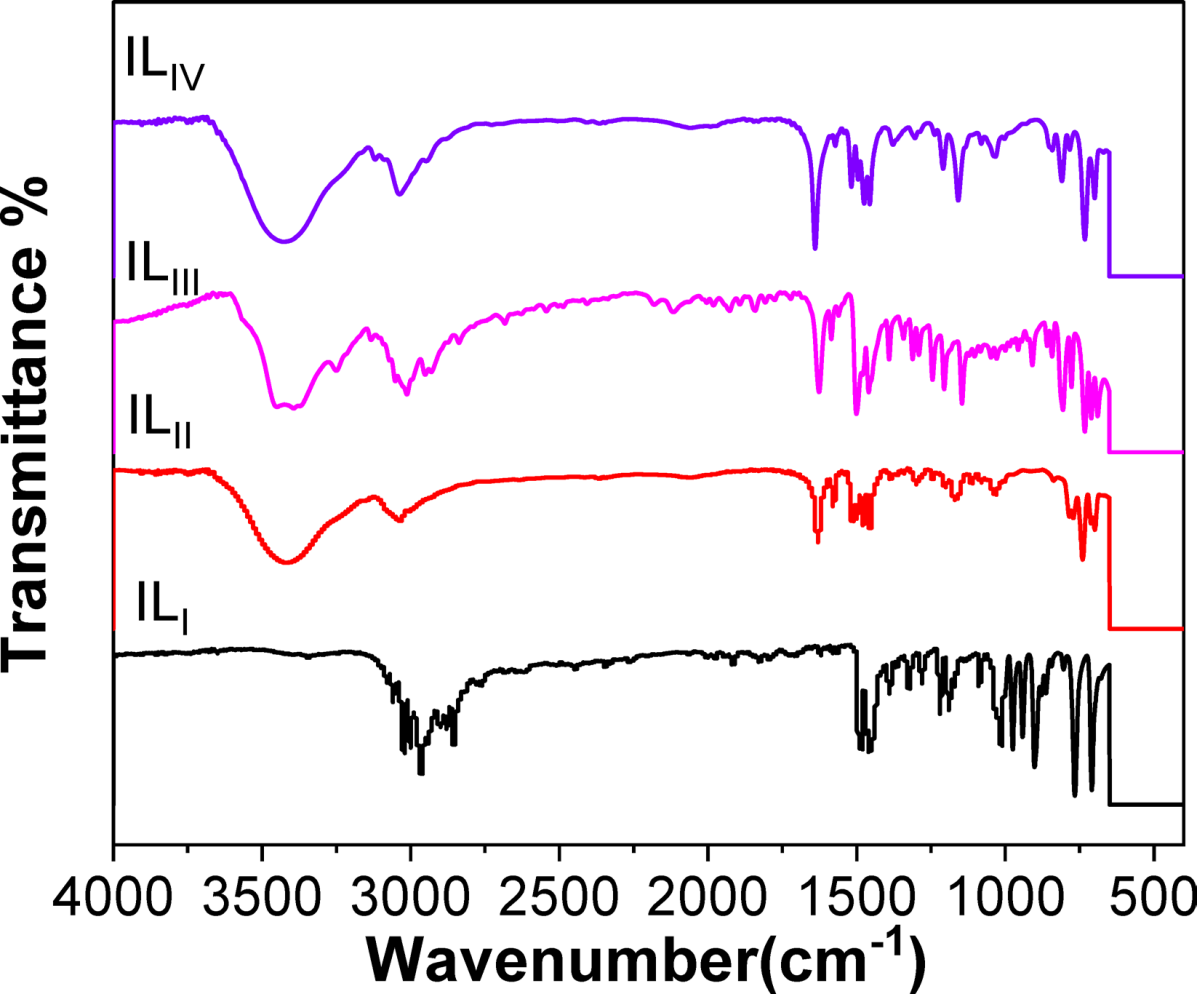
FTIR for ionic liquids IL_I_-IL_IV_.

Table 2 and Fig. 2 present the key IR Data about three distinct classes of compounds or chemical groups. FT-IR analysis was performed to verify the formation of the novel ILs, with the spectra shown in Fig. 2 and the recorded absorption bands summarized in Table 2. The absorption bands observed between 3339 and 3464 cm^− 1^ are ascribed to the stretching vibrations of hydrogen-bonded H₂O molecules. In the range of 3120–3170 cm^− 1^, the novel IL compound exhibits absorbance maxima associated with the stretching vibrations of C–H bonds in the aromatic ring. Additionally, the stretching vibrations of aliphatic C–H bonds are seen between 2855 and 2930 cm^− 1^. Bands in the 1383–1420 cm^− 1^ region are associated with C–N stretching modes in the piperidine and pyridine rings^36–38^.

**Table 2:** Infra-Red spectra of (IL_I_-IL_IV_) (wavenumber cm^-1^).

Cpd.	CH-H_2_O	C-H Aromatic	C-H aliphatic	C-*N* Aromatic
IL_I_	3344	3023-3002	2964-2855	1420
IL_II_	3420	3036	2988	1415
IL_III_	3393	3133-3012	2951-2837	11,425
IL_IV_	3424	3037	2921	1416

#### ^1^H-NMR

**Fig. 3:**
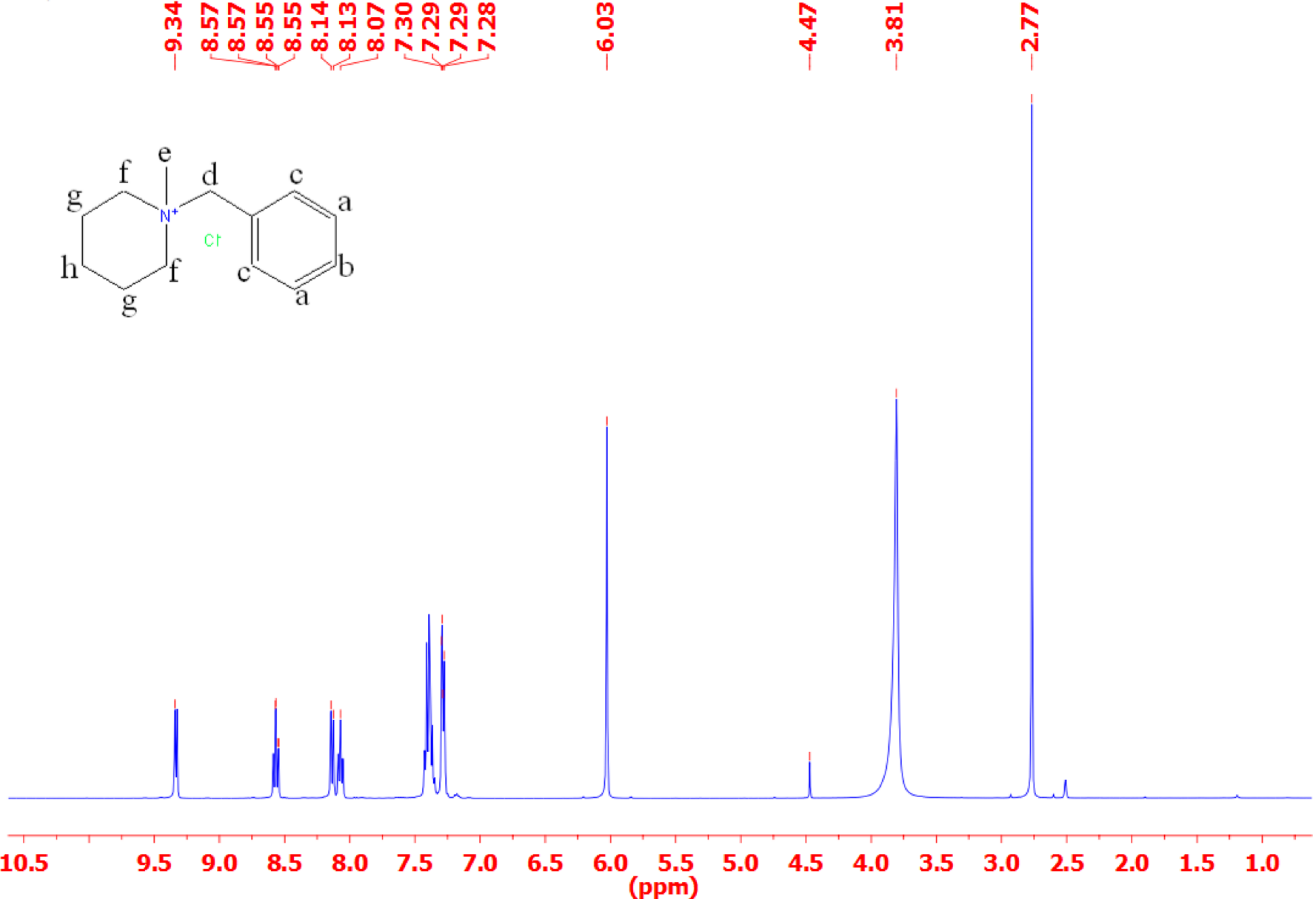
^1^HNMR for ionic liquid IL_I_.

**Fig. 4:**
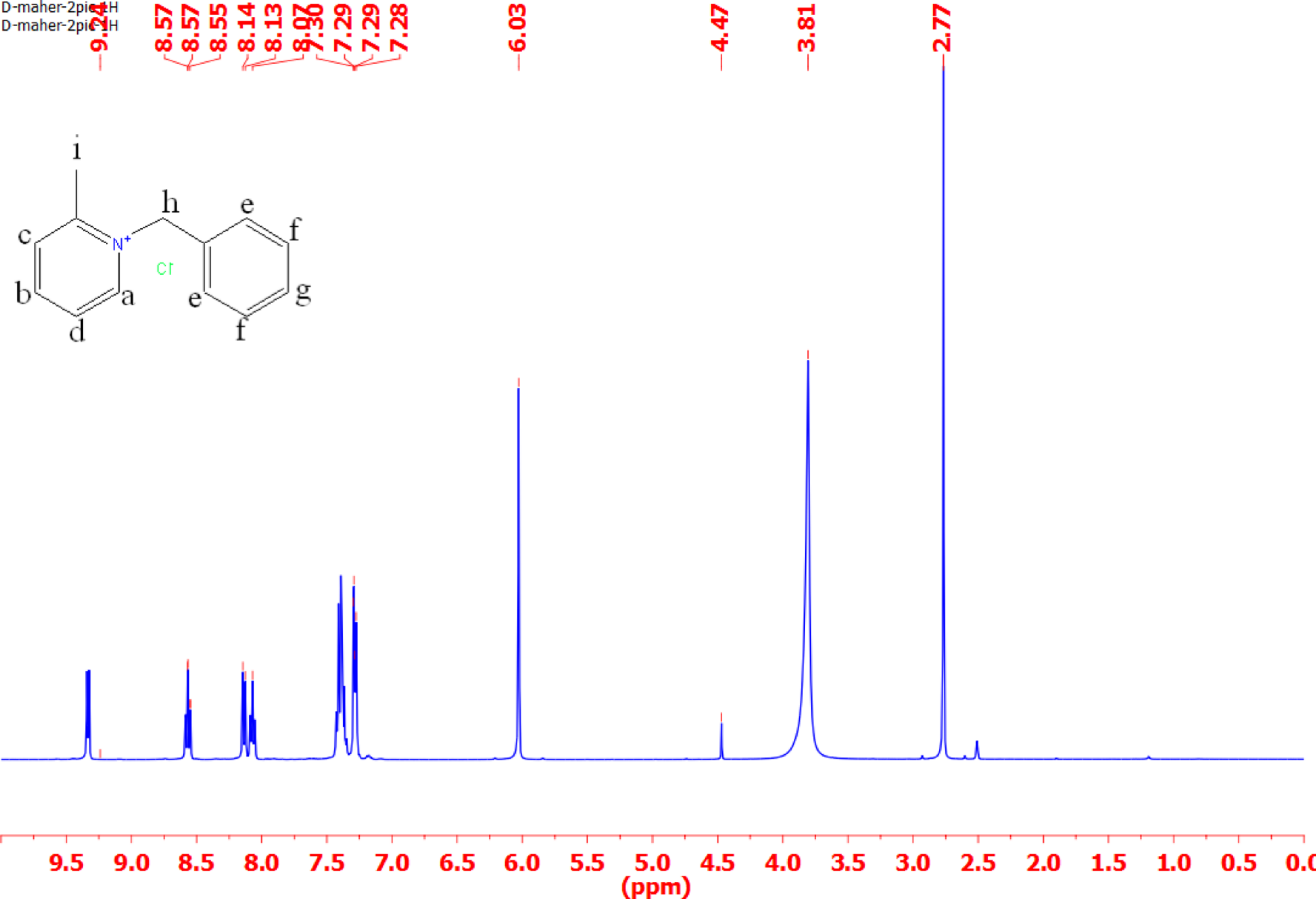
^1^HNMR for ionic liquid IL_II_.

**Fig. 5:**
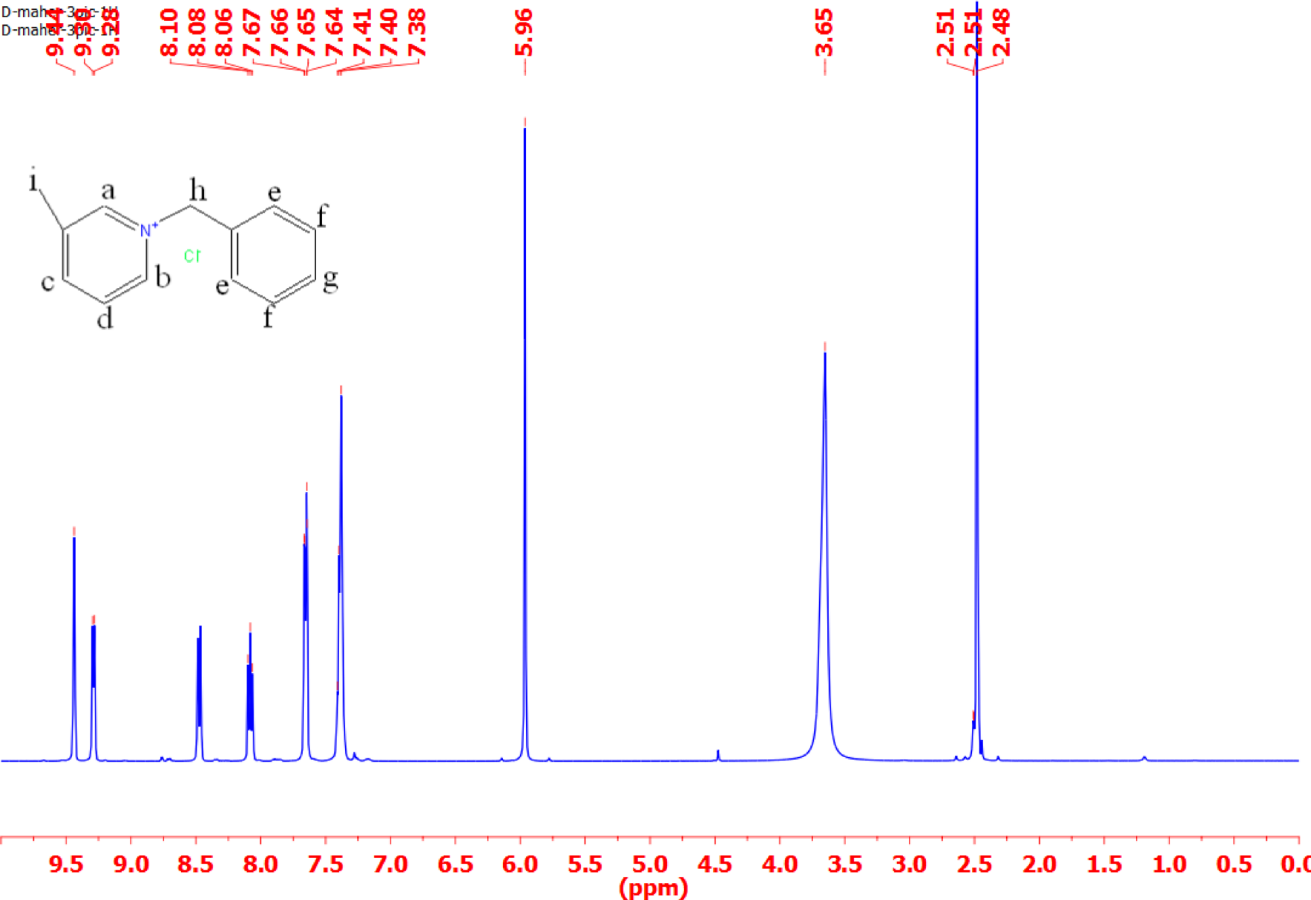
^1^HNMR for ionic liquid IL_III_.

**Fig. 6:**
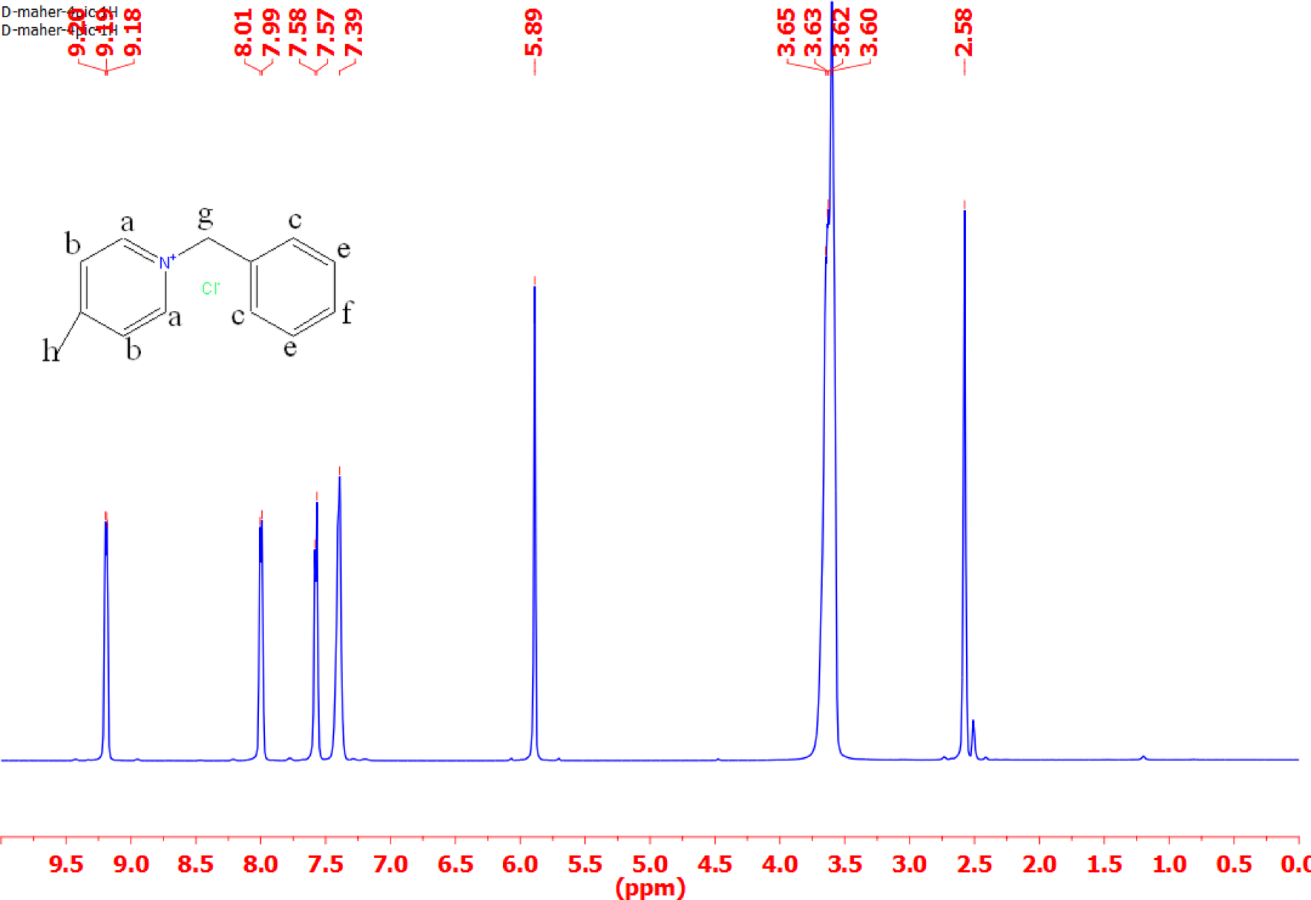
^1^HNMR for ionic liquid IL_IV_.

Tables 3 and Figs. 3, 4, 5 and 6 present the chemical shifts (δ ppm) observed in the proton nuclear magnetic resonance (NMR) spectra for the various compounds labeled as IL_I_, IL_II_, IL_III_, and IL_IV_. These chemical shifts provide insight into the local chemical environment of specific protons in each compound. For example, in compound IL_I_, protons ‘a,’ ‘b,’ ‘c,’ ‘d,’ ‘e,’ ‘f,’ ‘g,’ and ‘h’ exhibit distinct chemical shifts at 7.58, 7.55, 7.51, 4.65, 3.35, 1.87, 1.86, and 1.85 ppm, respectively. The corresponding spectral descriptions (e.g., ‘t’ for triplet, ‘d’ for doublet, ‘s’ for singlet) offer additional information about the proton’s coupling and multiplicity. Similarly, compounds IL_II_, IL_III_, and IL_IV_ display their respective chemical shifts and multiplicity patterns for their protons. These chemical shifts are essential for elucidating the molecular structure and connectivity of the compounds, assisting in their identification and structural characterization through NMR spectroscopy.

**Table 3:** Chemical shifts for different types of protons in IL_I_-IL_IV_.

Chemical shifts of different types of protons (δ ppm)									
Cpd.	a	b	c	d	e	f	g	h	i
IL_I_	7.58 (t)	7.55 (t)	7.51 (d)	4.65 (s)	3.35 (s)	1.87 (t)	1.86 (t)	1.85 (t)	-
IL_II_	9.24 (d)	8.75 (d)	8.14 (d)	8.07 (d)	7.3 (d)	7.29 (d)	7.28 (d)	6.03 (s)	3.81 (s)
IL_III_	9.44 (s)	9.3 (d)	8.1 (d)	8.08 (d)	7.66 (d)	7.41 (d)	7.38 (d)	5.96 (s)	3.65 (s)
IL_IV_	9.19 (d)	8.01 (d)	7.99 (d)	7.58 (d)	7.39 (d)	5.89 (s)	3.62 (s)	-	

### Electrochemical measurement

#### PP measurements

**Fig. 7:**
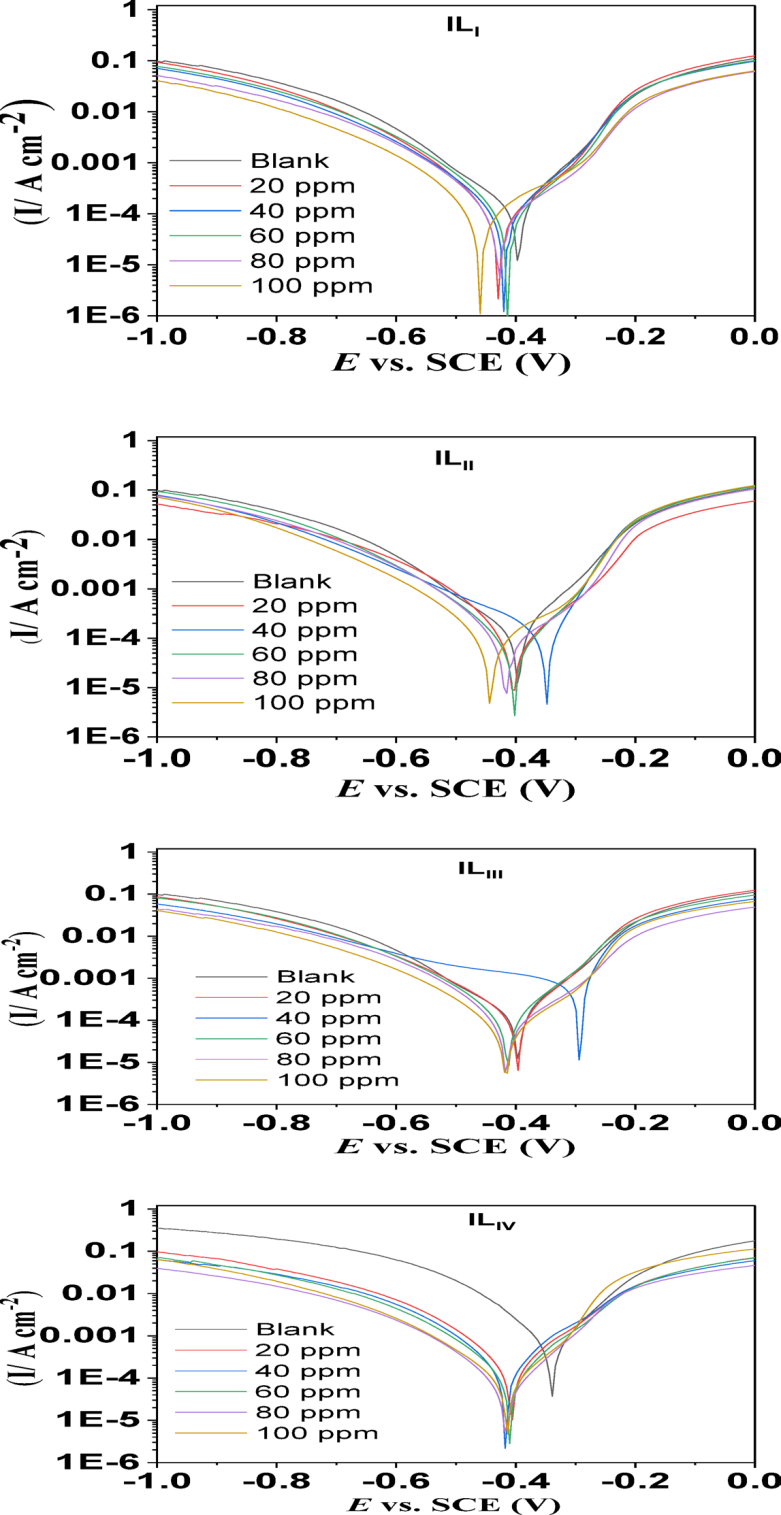
PP for ionic liquids (IL_I_-IL_IV_).

The plot of OCP against time for C-Steel in 1 M HCl solution in the absence and presence of different doses of ILs at 25 °C is shown in Fig. S2. After 900 s, the OCP became stable and steady values. Fig. 7 presents the Tafel polarization curves for C-steel in a 1 M (HCl) solution, both with and without inhibitors, at different concentrations of ILs at 25 °C. These curves provide insight into the electrochemical behavior of C-steel when exposed to the corrosive solution, with and without the presence of IL-based inhibitors. The polarization parameters associated with these curves are detailed in Table 4, including the current corrosion density (Icorr), which indicates the rate of electrochemical corrosion, as well as the Tafel slopes (βa and βc) that characterize the kinetics of the anodic and cathodic reactions, respectively. In addition to these parameters, the polarization resistance (Rp), which reflects the resistance of the material to corrosion, and the corrosion rate (C.R.), which quantifies the rate of material degradation, were also measured. Surface coverage (θ) was calculated, representing the fraction of the C-steel surface protected by the inhibitors, and the inhibitor efficiency (ηp%) was determined to evaluate how effectively the ILs prevent corrosion. As the concentration of ILs increases, a significant decrease in the corrosion current density (I_corr_) is observed, which implies that the ILs are successful in reducing the rate of corrosion of C-steel in the HCl solution. This reduction in Icorr suggests that, at the optimal concentration, the ILs function effectively as corrosion inhibitors. Furthermore, both the anodic and cathodic branches of the polarization curves shift toward more negative current densities with increasing IL concentration. This shift indicates that the inhibitor acts in a mixed inhibition mode, simultaneously affecting both anodic and cathodic reactions. In other words, the inhibitor not only slows down the anodic dissolution of the steel but also reduces the cathodic reactions, thereby significantly decreasing the overall corrosion rate^39,40^. Furthermore, the adsorption of ILs onto the steel surface, which blocks the active sites, does not alter the underlying corrosion reaction mechanism^41^. The θ and ηp% were determined using the following Eqs.^42,43^:

**Table 4:** Polarization parameters for IL_I_-IL_IV_ in 1 M HCl without and with various concentrations.

Cpd.	Conc. (M )	-Eco. (mV) vs. SCE	Ico. (μA cm^-2^)	βa (mV dec^-1^)	βc (mV dec^-1^)	k (m.*p*.y)	θ	ηp %
Blank	0 ppm	339	4180	313.1	350	1911	-	
IL_I_	20 ppm	429	248	186.5	258.0	113.3	0.9407	94.07
	40 ppm	420	240	156.4	232.6	109.9	0.9426	94.26
	60 ppm	414	202	142.0	214.4	92.51	0.9517	95.17
	80 ppm	427	162	159.3	217.3	74.23	0.9612	96.12
	100 ppm	460	156	173.5	226.5	71.44	0.9627	96.27
IL_II_	20 ppm	403	272	202.6	272.0	124.5	0.9349	93.49
	40 ppm	349	230	109.7	249.5	105.3	0.9449	94.49
	60 ppm	402	133	121.0	190.6	60.85	0.9681	96.81
	80 ppm	416	122	132.0	199.3	55.81	0.9708	97.08
	100 ppm	442	105	131.6	190.8	48.11	0.9748	97.48
IL_III_	20 ppm	397	284	129.6	224.1	129.9	0.9321	93.21
	40 ppm	416	304	252.9	321.3	138.7	0.9273	92.73
	60 ppm	413	289	154.4	227.1	132.0	0.9309	93.09
	80 ppm	416	237	207.4	277.3	108.3	0.9433	94.33
	100 ppm	416	164	189.2	290.7	80.31	0.9579	95.79
IL_IV_	20 ppm	407	783	255.8	299.2	357.6	0.8127	81.27
	40 ppm	418	453	181.2	243.9	207.1	0.8916	89.16
	60 ppm	410	418	195.0	276.4	190.9	0.9012	90.12
	80 ppm	416	187	164.4	247.7	85.52	0.9553	95.53
	100 ppm	414	122	97.6	176.3	55.87	0.9708	97.08

1 $$\Theta {\text{ }}={\text{ }}\left[ {\left( {{{\mathrm{I}}_{0{\text{ corr}}}}-{\text{ }}{{\mathrm{I}}_{{\mathrm{corr}}}}} \right)/{{\mathrm{I}}_0}{\mathrm{corr}}} \right]$$

2 $${\mathrm{\boldsymbol{\upeta}p}}\% {\text{ }}={\text{ }}\left[ {\left( {{{\mathrm{I}}_{0{\text{ corr}}}}-{\text{ }}{{\mathrm{I}}_{{\mathrm{corr}}}}} \right)/{{\mathrm{I}}_{0{\mathrm{corr}}}}} \right] \times {\text{ 1}}00$$

where I_corr_ and I_0 corr_ represent the corrosion current densities in the presence and absence of the inhibitor, respectively, after calculating ηp%, we found that the inhibition efficiency of the ILs was approximately 96%.

#### EIS measurements

**Fig. 8:**
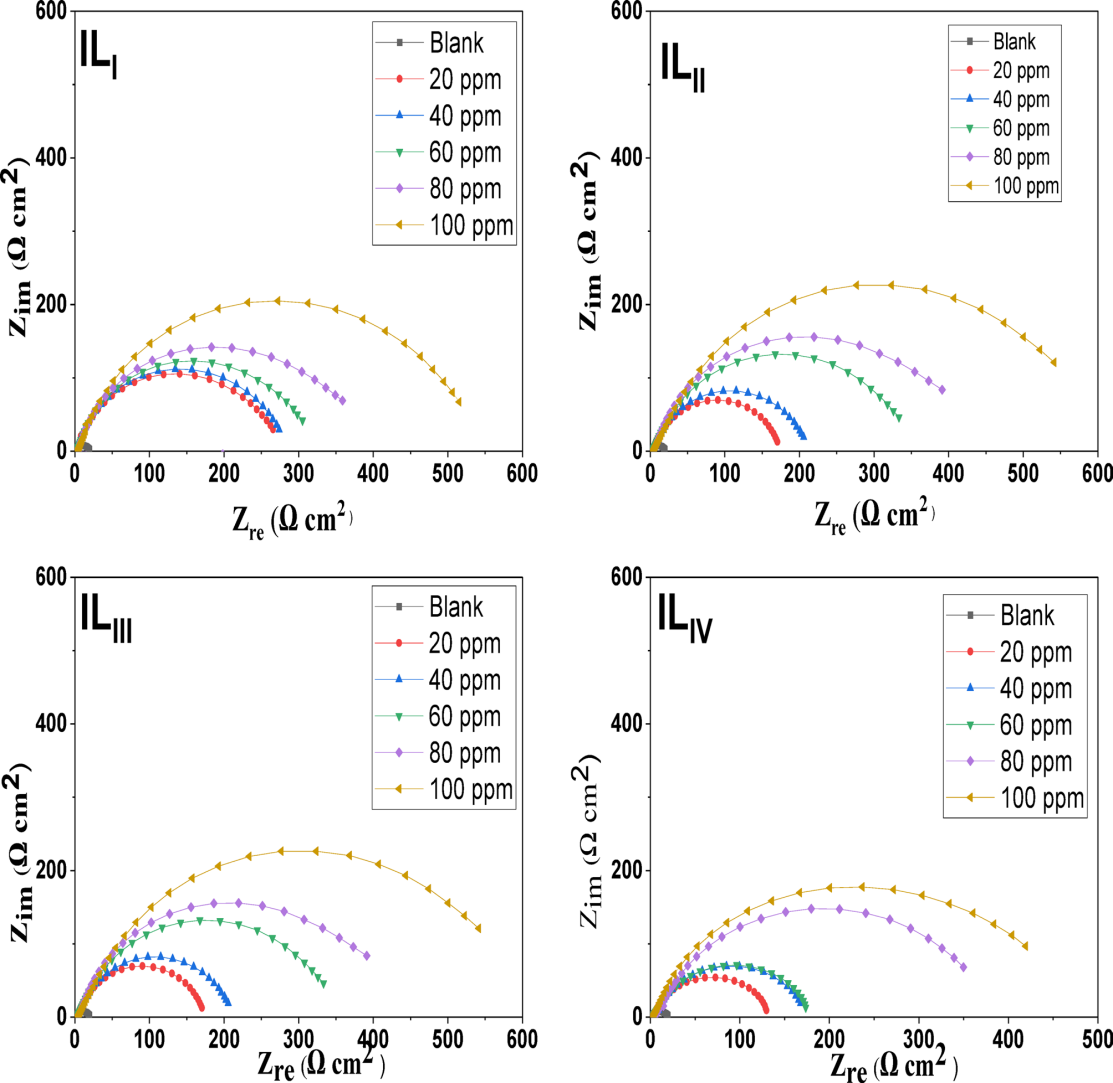
Nyquist plots for ionic liquids (IL_I_-IL_IV_).

**Fig. 9:**
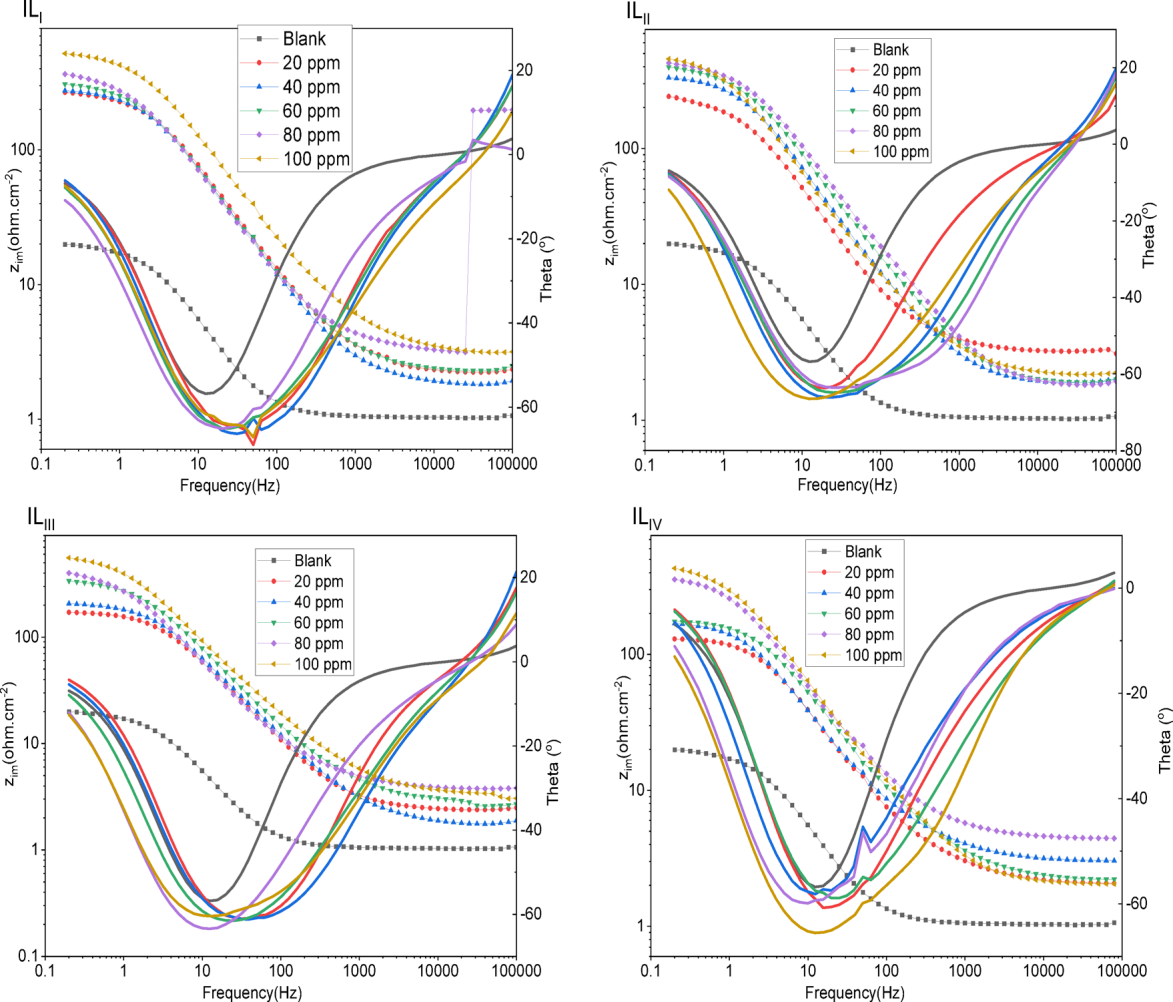
Bode plots for ionic liquids (IL_I_-IV).

**Fig. 10:**
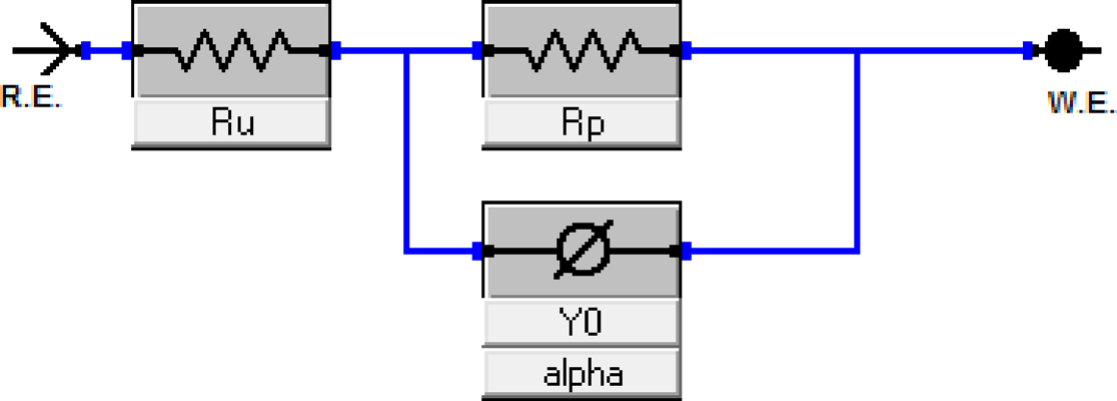
Equivalent circuit used for EIS analysis.

EIS offers crucial information regarding the dynamics of electrode processes as well as the surface properties of the system being studied. The patterns observed in the Nyquist plots reveal important mechanistic details about the interactions occurring at the electrode interface^44,45^. Fig. 8 displays the Nyquist plots for ILs in a 1 M HCl solution at 25 °C, showing data for both the absence and presence of different concentrations of the inhibitors. The impedance parameters obtained from the EIS measurements are summarized in Table 5. The Nyquist plots clearly indicate that the presence of the inhibitors significantly influences the impedance response of mild steel in the acidic solution. This observation suggests that as the concentration of the inhibitors increases, the impedance of the solution also rises. The plots display a single depressed semicircle common to both the uninhibited and inhibitor-containing solutions. However, the diameter of this semicircle expands considerably with higher inhibitor concentrations, reflecting a shift in impedance. This increase in diameter with rising IL concentrations indicates that the corrosion mechanism remains unchanged with the addition of the inhibitors^46^. The observed rise in impedance at low frequencies (Fig. 9) can be attributed to the adsorption of inhibitor compounds onto the surface of C-steel. To analyze the impedance spectra of mild steel corrosion in 1 M HCl, both with and without various concentrations of the inhibitor compounds, the Randle equivalent circuit was applied to model the data (Fig. 10). Furthermore, a single maximum in the Bode plots (Fig. 9) suggests that a single charge transfer primarily hindered the corrosion process^47,48^. The inhibition efficiency improves as the inhibitor concentration increases, with the highest concentration of 100 ppm showing a maximum inhibition efficiency of approximately 96%. The (ηp %) was determined by the following equations^49^:

**Table 5:** EIS parameters for ILI-ILIV in 1 M HCl without and with various concentrations.

Cpd.	Conc. (M)	*R*_s_ (*R*_u_) (Ω cm^2^)	*R*_ct_ (*R*_*p*_) (Ω cm^2^)	Y_o_ (μ Ω ^-1^ s^*n*^ cm^-2^) X10^-3^	*n*	chi squaredX10^-3^	θ	ηp %
Blank	Blank	1.038	18.87	4.477	0.899	0.352	-	-
IL_I_	20 ppm	2.298	278.2	0.392	0.824	5.50	0.9322	93.22
	40 ppm	1.864	288.9	0.402	0.832	6.25	0.9347	93.47
	60 ppm	2.339	329.8	0.440	0.8.11	5.75	0.9428	94.28
	80 ppm	3.36	387.6	0.454	0.819	1.02	0.9513	95.13
	100 ppm	3.263	570.1	0.277	0.786	4.65	0.9669	96.69
IL_II_	20 ppm	3.3	240.5	0.584	0.836	4.20	0.9215	92.15
	40 ppm	1.897	360.8	0.429	0.820	7.77	0.9477	94.77
	60 ppm	1.881	425.1	0.367	0.798	6.52	0.9556	95.56
	80 ppm	1.781	456.7	0.327	0.791	7.50	0.9587	95.87
	100 ppm	2.23	534.5	0.512	0.799	7.16	0.9647	96.47
IL_III_	20 ppm	2.403	177.7	0.497	0.824	5.14	0.8938	89.38
	40 ppm	1.787	220.5	0.522	0.792	8.10	0.9144	91.44
	60 ppm	2.806	371.5	0.472	0.776	8.05	0.9492	94.92
	80 ppm	3.915	441. 7	0.590	0.801	0.439	0.9572	95.72
	100 ppm	3.359	676.2	0.448	0.747	530	0.9721	97.21
IL_IV_	20 ppm	2.18	142.7	0.89	0.79	4.95	0.8678	86.78
	40 ppm	3.177	191.1	0.99	0.76	4.82	0.9013	90.13
	60 ppm	2.284	194.5	0.72	0.76	4.12	0.9030	90.30
	80 ppm	4.563	421.4	0.66	0.76	3.09	0.9552	95.52
	100 ppm	2.062	507.4	0.56	0.78	2.88	0.9628	96.28

3 $$\Theta {\text{ }}={\text{ }}[({{\mathrm{R}}_{{\mathrm{oct}}}} - {{\mathrm{R}}_{{\mathrm{ct}}}})/{{\mathrm{R}}_{{\mathrm{oct}}}}]$$

4 $${\mathrm{\boldsymbol{\upeta}p}}\% ={\text{ }}[({{\mathrm{R}}_{{\mathrm{oct}}}} - {{\mathrm{R}}_{{\mathrm{ct}}}})/{{\mathrm{R}}_{{\mathrm{oct}}}}] \times {\text{ 1}}00$$

where R_ct_ and R_oct_ represent the charge transfer resistances with and without inhibitors, respectively. The inhibition efficiency increases with higher inhibitor concentrations, reaching its peak at the maximum concentration.

#### EFM measurements

**Fig. 11:**
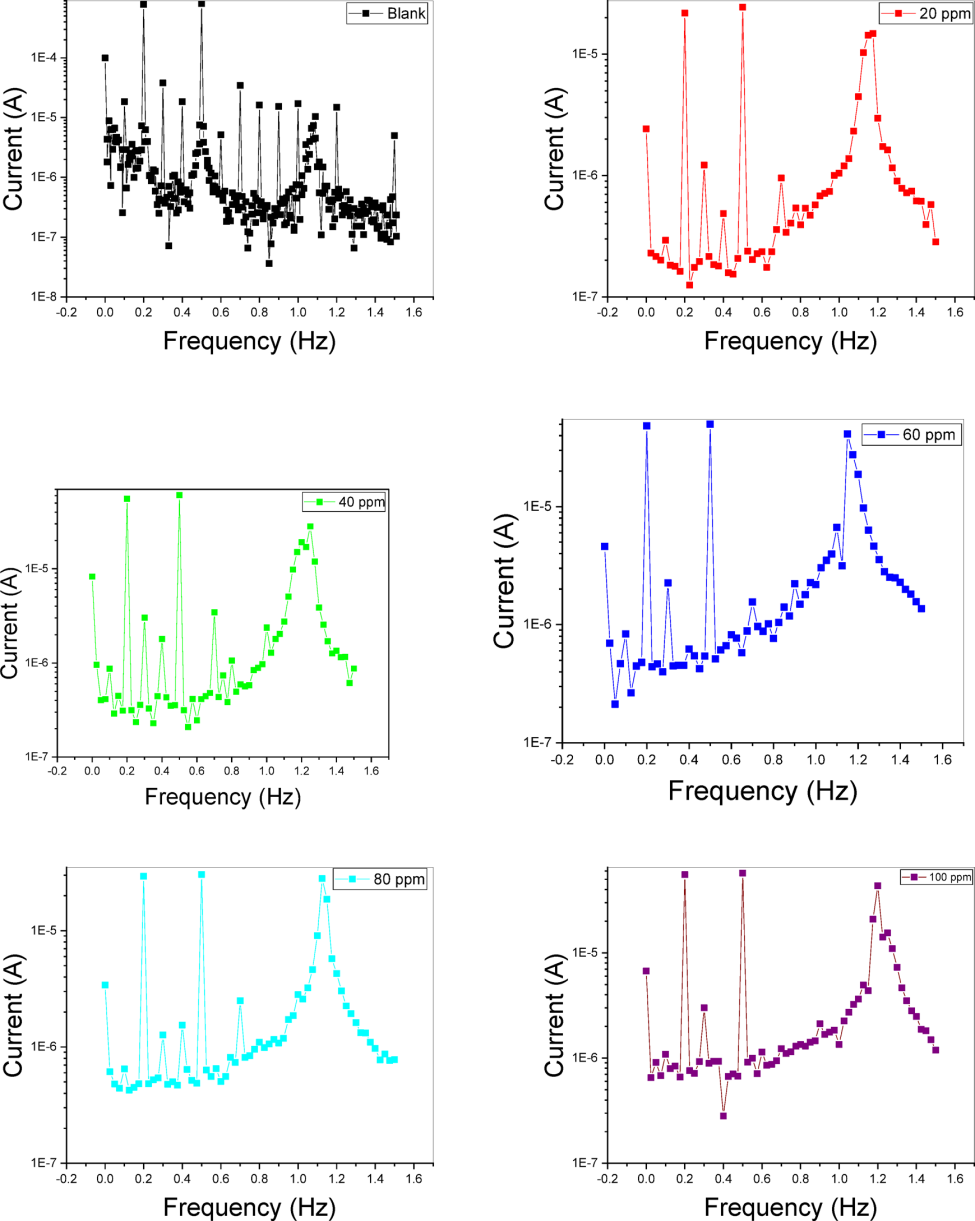
EFM for IL_I_.

Fig. 11 presents a representative electrochemical frequency modulation (EFM) spectrum for C-steel in 1 M HCl at 25 °C for ILI, illustrating the harmonic and intermodulation current peaks. These prominent peaks were utilized to determine the Icorr, Tafel slopes (βc and βa) and causality factors (CF-2 and CF-3), with the extracted values summarized in Table 6. The corrosion rate of C-steel declines as the inhibitor concentration increases. A significant reduction in the C.R. from approximately 460 m.p.y to 11.40 m.p.y for ILIV demonstrates the high efficiency of the corrosion inhibitors studied. At the highest concentration, the inhibition efficiency reaches approximately 97%.

**Table 6:** EFM parameters for IL_I_-IL_IV_ in 1 M HCl without and with various concentrations.

Cpd.	Conc. (M)	Icorr (μA cm^-2^ )	βa (mV dec^-1^ )	βc (mV dec^-1^ )	CF-2	CF-3	k (m.*p*.y)	θ	I_EFM_ %
Blank	0 ppm	1007	73.76	92.82	2.033	3.191	460	-	-
IL_I_	20 ppm	37.35	42.83	44.57	1.920	3.292	17.07	0.9629	96.29
	40 ppm	33.85	35.6	40.52	1.543	3.512	15.47	0.9663	96.63
	60 ppm	25.74	32.7	35.50	1.359	2.918	11.76	0.9744	97.44
	80 ppm	21.89	43.73	52.65	1.320	2.77	10.00	0.9782	97.82
	100 ppm	21.57	24.27	25.7	1.521	2.593	9.858	0.9785	97.85
IL_II_	20 ppm	66.74	89.42	144.5	1.883	1.845	30.50	0.9337	93.37
	40 ppm	49.74	64.58	78.76	1.678	4.011	22.73	0.9506	95.06
	60 ppm	48.35	66.67	96.79	2.076	4.953	22.09	0.9519	95.19
	80 ppm	44.29	84.65	98.97	1.996	2.626	20.24	0.9560	95.60
	100 ppm	42.24	65.31	89.71	1.682	2.749	19.30	0.9581	95.81
IL_III_	20 ppm	122.8	89.91	93.7	2.37	3.198	56.12	0.8780	87.80
	40 ppm	101.4	86.10	93.60	2.552	2.95	46.32	0.8993	89.93
	60 ppm	60.86	84.83	101.6	2.162	3.413	27.81	0.9396	93.96
	80 ppm	56.18	92.49	97.37	1.159	2.628	25.67	0.9442	94.42
	100 ppm	46.51	106.8	131.9	2.23	2.543	21.25	0.9538	95.38
IL_IV_	20 ppm	89.97	50.37	51.86	4.172	3.107	41.11	0.9106	91.06
	40 ppm	89.89	65.65	71.92	1.35	3.41	41.08	0.9107	91.07
	60 ppm	67.09	47.51	50.34	4.606	2.357	30.65	0.9333	93.33
	80 ppm	31.92	48.99	64.72	2.08	10.1	14.59	0.9683	96.83
	100 ppm	24.14	53.9	64.77	11.87	6.85	11.03	0.9760	97.60

Based on the data obtained from PP, EIS, and EFM, the four IL compounds (ILI-IV) effectively inhibit the steel erosion of 1 M HCl at a concentration of 100 ppm. The primary structural difference among these compounds lies in the number of aromatic rings and the position of methyl substitution, with all four compounds possessing nitrogen atoms with lone pairs of electrons. ILI, containing only a single aromatic ring, exhibits lower inhibition efficiency compared to ILII-IV. This can be related to the greater ability of multiple aromatic rings to donate e- to the vacant d-orbitals of Fe while simultaneously accepting electrons from Fe, thereby enhancing adsorption and corrosion inhibition (See supplementary fig.S3)^47–49^.

### Adsorption isotherm

Isotherms are essential tools for characterizing the mode and extent of these interactions. In this research, adsorption is linked to the θ of the metal by the inhibitor and its concentration in the corrosive medium. A range of adsorption isotherm models including Langmuir, Freundlich, Temkin, Frumkin, and Flory-Huggins were applied to fit the potentiodynamic polarization data. The linear equations, along with the corresponding slope values, intercepts, and regression coefficients (R²), are summarized in Table 7. Among these models, the Langmuir isotherm provided the best fit, as indicated by R² values closest to unity, suggesting monolayer adsorption of the ILs on the C-steel surface^50^. The adsorption of ILs onto the mild steel surface is best represented by the Langmuir adsorption isotherm, which is mathematically described by the following expression^50^:

**Table 7:** Adsorption isotherm models for ILI–ILIV inhibitors, including R² values, slopes, and intercepts, based on data from potentiodynamic polarization measurements at 25 °C.

Adsorption isotherm		Flory-Huggins isotherm	Freundlich isotherm	Frumkin isotherm	Langmuir isotherm	Temkin isotherm
Equation		log(θ/C) = log K + n log (1-θ)	log θ = log k + 1/n log C	log θ/(1−θ) C = log k+ 2a θ	$\:\frac{{\boldsymbol{C}}_{\:}\:}{\boldsymbol{\theta\:}}=\frac{1}{{\boldsymbol{K}}_{\:}\:}+\:{\boldsymbol{C}}_{\:}$	$\:\boldsymbol{\uptheta\:}=-\frac{1}{{2\boldsymbol{a}}_{\:}\:}\mathbf{ln}\boldsymbol{C}\:-\frac{1}{{2\boldsymbol{a}}_{\:}\:}\mathbf{ln}\boldsymbol{K}$
IL_I_	R^2^	0.9133	0.9969	0.9150	0.9999	0.8488
	Slope	1.3872	-0.0498	-3.7848	1.0301	-2.8739
	intercept	-0.0520	0.0761	10.4277	0.9628	16.7627
IL_II_	R^2^	0.9125	0.9915	0.9724	0.9999	0.9228
	Slope	1.3691	-0.0441	-2.5046	1.0123	-1.5288
	intercept	-0.0317	0.0640	7.7874	1.4011	10.4580
IL_III_	R^2^	0.9176	0.9943	0.7062	0.9996	0.5077
	Slope	1.3918	-0.0589	-3.0760	1.0377	-2.1447
	intercept	-0.0517	0.0937	9.2532	1.3854	14.0048
IL_IV_	R^2^	0.9051	0.9137	0.9705	0.9986	0.8594
	Slope	1.2511	-0.0903	-1.8918	0.9784	-0.8718
	intercept	0.1458	0.1519	6.8873	5.8572	8.2135

5 $$\:\frac{c}{\theta\:}=\:\frac{1}{\mathrm{k}}+\:c$$

where K_ads_ is the equilibrium constant of the adsorption process, and C represents the inhibitor concentration. By substituting the experimentally obtained θ values from PP measurements into this equation and plotting C/θ against C, K_ads_ can be determined, as shown in Fig. 12.

**Fig. 12:**
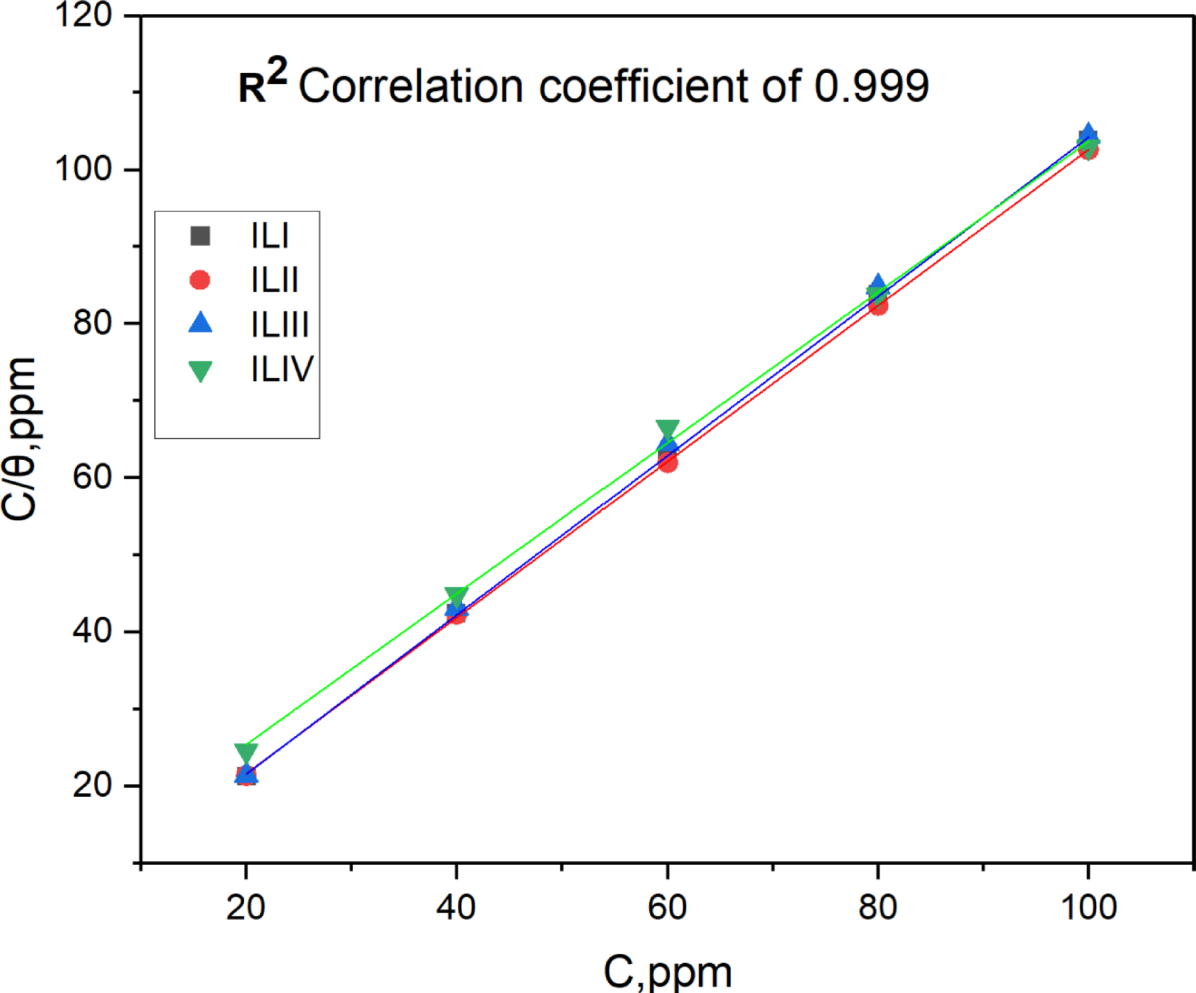
Langmuir adsorption for ILs at 25 °C. Obtained from pp data.

The calculation of the standard free energy of adsorption (ΔG°ads) was carried out using the following equation^51^:

6 $$\Delta {{\mathrm{G}}^0}{\mathrm{ads}}\,=\, - \,{\text{RT ln }}\left( {{\mathrm{55}}.{\text{5 }}{{\mathrm{K}}_{{\mathrm{ads}}}}} \right)$$

In this equation, the constant 55.5 corresponds to the molar concentration of water in the solution, R denotes the universal gas constant (8.314 J K^− 1^ mol^− 1^), and T represents the absolute temperature.

The calculated values of ΔG°ads are − 34.30, − 33.38, − 33.41, and − 29.89 kJ·mol⁻¹ for IL_I_, IL_II_, IL_III_, and IL_IV_, respectively. The adsorption process can be categorized based on the values of ΔG°ads as follows:

(i) When ΔG°ads exceeds − 20 kJ mol⁻¹, the adsorption is mainly due to physical interactions (physisorption), which are governed by electrostatic forces.
(ii) When ΔG°ads is below − 40 kJ mol⁻¹, the adsorption is primarily chemical (chemisorption), involving the formation of bonds through electron transfer from the inhibitor to the metal surface.
(iii) For values of ΔG°ads between − 20 and − 40 kJ mol⁻¹, a combination of both physical and chemical adsorption mechanisms occurs.

For the ILs studied here, the computed ΔG°ads values fall below − 40 kJ mol⁻¹, indicating that the adsorption mechanism is a blend of both physical and chemical adsorption^52–55^.

### Quantum chemical calculations

**Fig. 13:**
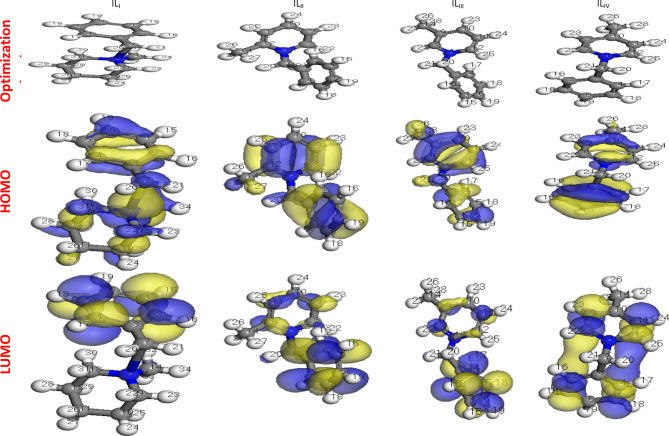
Optimized structures, HOMO and LUMO distributions of IL_I_-IL_IV_.

**Fig. 14:**
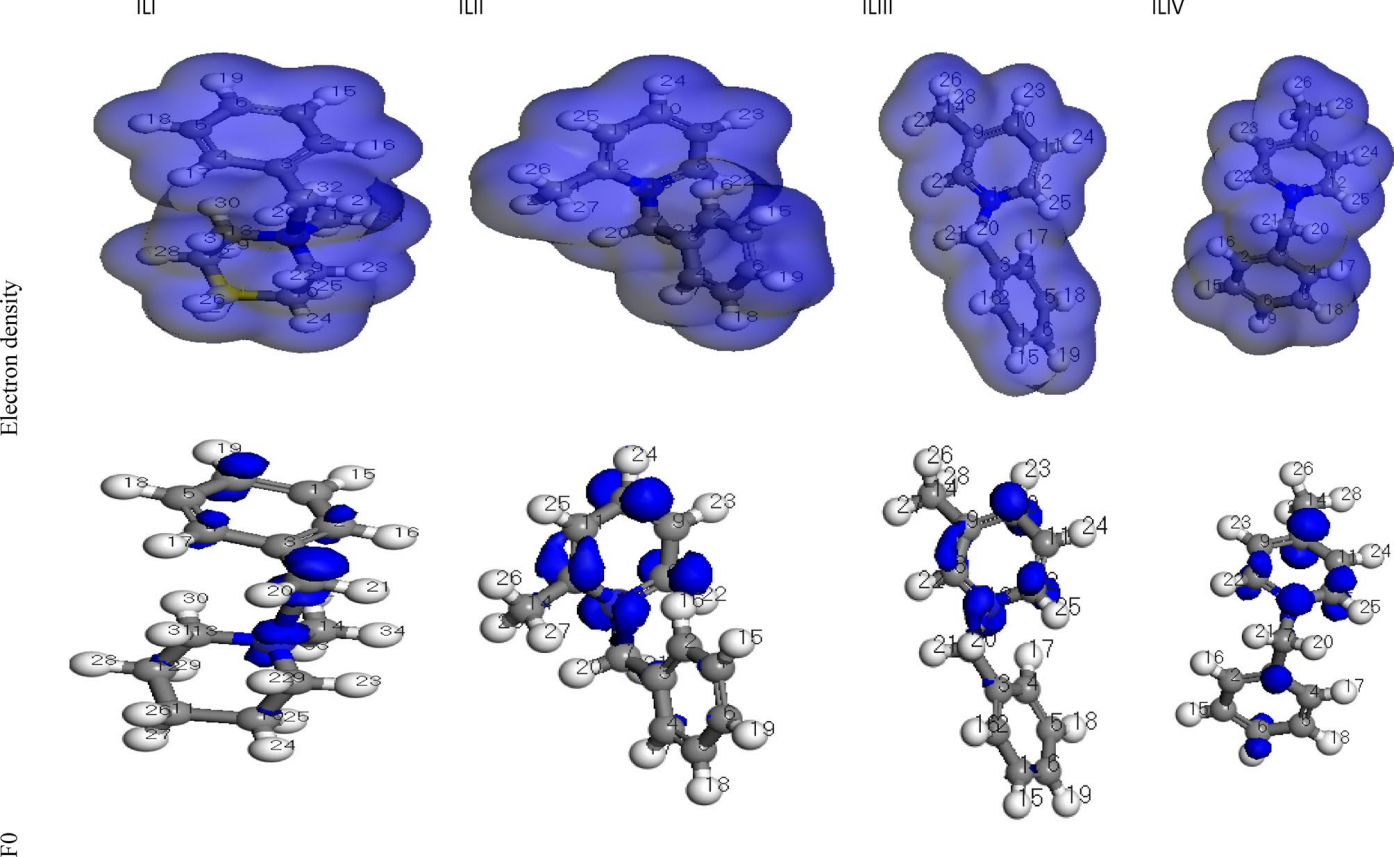
Electron density and Muliken charge for ILs.

Figs. 13 and 14 illustrate the optimized geometry structure, along with the HOMO, LUMO, and electron density distribution of the cationic inhibitor. The HOMO (Highest Occupied Molecular Orbital) signifies the ability of the inhibitor molecule to release electrons. In contrast, the LUMO (Lowest Unoccupied Molecular Orbital) represents its capacity to receive electrons from the metal surface^53^. The energy values of HOMO and LUMO correlate with parameters such as work function (Ø), chemical hardness (h), global softness (S), and electrophilicity index (ω). Table 8 presents the Fukui indices and Mulliken atomic charges for the four synthesized ionic liquids (IL_I_–IL_IV_)^28^ .

**Table 8:** Calculated quantum-chemical parameters for the synthesized Azo phenol compounds IL_I_-IL_IV_.

Chemical Parameter*	IL_I_	IL_II_	IL_III_	IL_IV_
E_HOMO_	-0.1079	-0.0739	-0.0771	-0.0767
E_LUMO_	-0.0271	-0.0506	-0.0483	-0.0507
ΔE _Gap_	0.0809	0.0234	0.0288	0.0261
I	0.1079	0.0739	0.0771	0.0767
A	0.0271	0.0506	0.0483	0.0507
X	0.0675	0.0623	0.0627	0.0637
h	0.04043	0.0117	0.0144	0.0131
*S*	24.7338	85.4810	69.3722	76.6577
w	0.0564	0.1658	0.1364	0.1557
ΔE _Back-donation_	0.0101	0.0029	0.0036	0.0033
ΔN	87.4032	301.8459	244.9779	270.7449

All parameters in eV except ΔN is a dimensionless quantity.

**Fig. 15:**
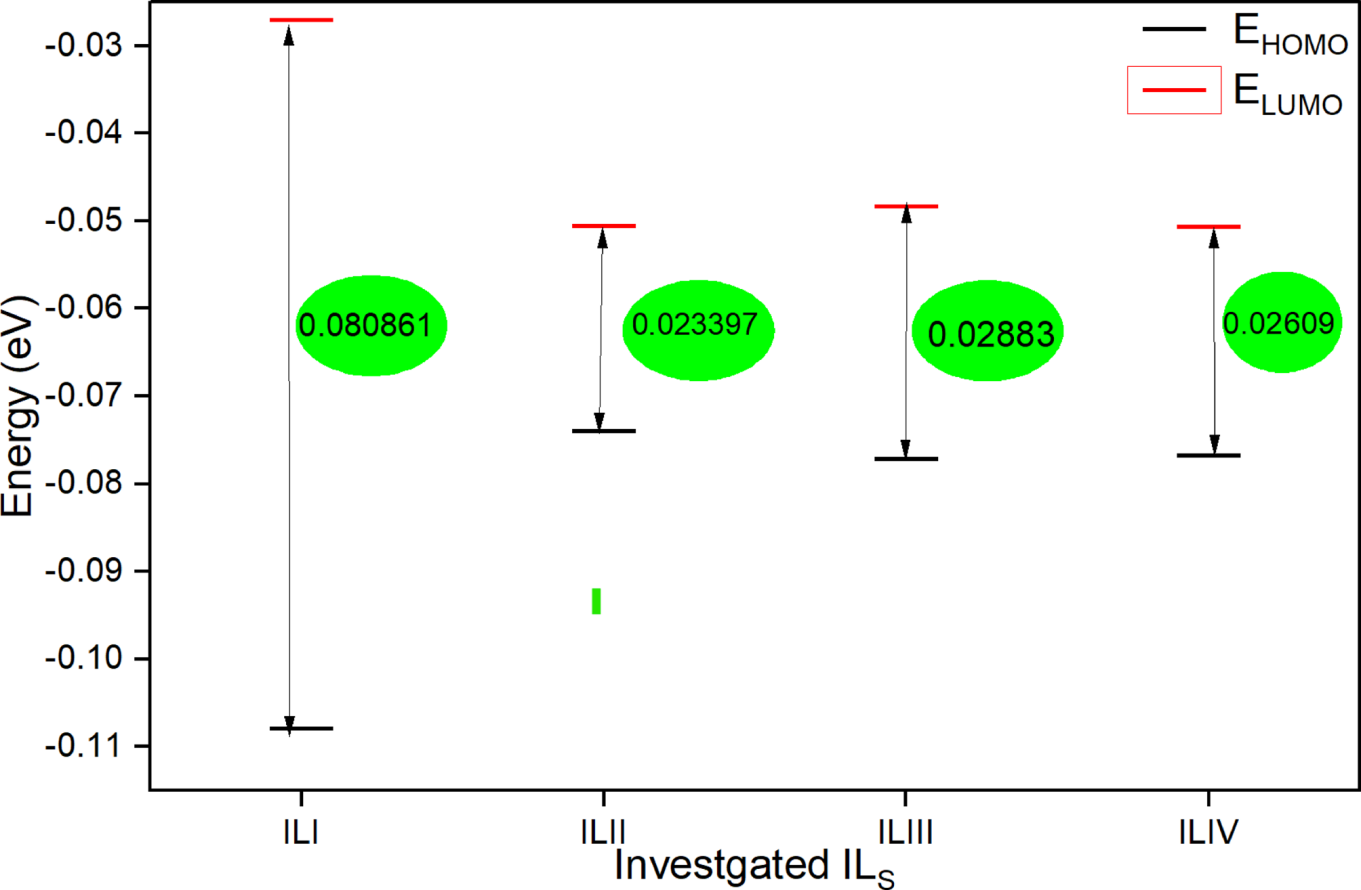
ΔE values for compound IL_I_-IL_IV_.

According to frontier molecular orbital theory, an inhibitor molecule with a higher E_HOMO_ value can more readily transfer electrons to the vacant d-orbitals of iron. Conversely, a lower E_LUMO_ value enhances the molecule’s ability to accept electrons from Fe. A smaller energy gap (ΔE = E_LUMO_ - E_HOMO_) generally indicates a higher adsorption capability of the inhibitor onto the surface of C-steel, enhancing its corrosion inhibition potential^56,57^. As shown in Table 9; Fig. 15, the ΔE values for IL_II_–IL_IV_ are lower compared to IL_I_. This suggests that IL_II_–IL_IV_, which contains two aromatic rings, can more effectively donate and accept electrons in interactions with Fe. Among them, ILII exhibits the highest adsorption strength on C-steel and superior inhibition performance relative to the other ionic liquids. These findings align well with the experimental data, further confirming the theoretical calculations. Table 10 presents a comparative analysis of the inhibition efficiency (IE%) of various ionic liquids (ILs) used as corrosion inhibitors for carbon steel in acidic media, specifically at relatively high concentrations. The inhibition efficiencies were determined using Electrochemical Impedance Spectroscopy (EIS), a reliable technique for evaluating corrosion protection performance^58,59^.

**Table 9:** Calculated Fukui indices and mulliken atomic charges for IL_I_-IL_IV_.

IL_I_				IL_II_				IL_III_				IL_IV_			
**Atom**	**F0**	**f+**	**f-**	Atom	**F** ^**0**^	**f** ^**+**^	**f** ^**-**^	Atom	**F** ^**0**^	**f** ^**+**^	**f** ^**-**^	Atom	**F** ^**0**^	**f** ^**+**^	**f** ^**-**^
C (1)	0.008	0.008	0.008	C (1)	0.017	0.019	0.014	C (1)	0.02	0.024	0.016	C (1)	0.009	0.009	0.009
C (2)	0.035	0.033	0.037	C (2)	-0.011	-0.01	-0.011	C (2)	0.006	0.006	0.006	C (2)	0.004	0.008	0.001
C (3)	-0.015	-0.01	-0.015	C (3)	-0.008	-0.006	-0.009	C (3)	-0.018	-0.014	-0.022	C (3)	0.005	0.005	0.005
C (4)	0.036	0.037	0.036	C (4)	0.012	0.016	0.009	C (4)	0.005	0.01	0.001	C (4)	0.009	0.009	0.008
C (5)	0.007	0.007	0.008	C (5)	0.008	0.008	0.008	C (5)	0.016	0.018	0.014	C (5)	0.006	0.006	0.006
C (6)	0.063	0.062	0.063	C (6)	0.015	0.016	0.013	C (6)	0.008	0.007	0.01	C (6)	0.032	0.034	0.03
C (7)	0.09	0.09	0.09	C (7)	-0.027	-0.027	-0.027	C (7)	-0.039	-0.04	-0.038	C (7)	-0.031	-0.029	-0.033
N (8)	-0.002	-0	-0.001	C (8)	0.084	0.082	0.087	C (8)	0.058	0.052	0.065	C (8)	0.045	0.047	0.043
C (9)	-0.025	-0.03	-0.025	C (9)	-0.001	-0.002	0	C (9)	-0.002	-0.001	-0.004	C (9)	0.012	0.006	0.019
C (10)	-0.018	-0.02	-0.019	C (10)	0.104	0.099	0.108	C (10)	0.104	0.1	0.107	C (10)	0.064	0.061	0.067
C (11)	-0.016	-0.02	-0.015	C (11)	0.015	0.015	0.015	C (11)	0	-0.001	0.001	C (11)	0.006	0.008	0.004
C (12)	-0.015	-0.02	-0.015	C (12)	0.036	0.031	0.041	C (12)	0.086	0.083	0.089	C (12)	0.06	0.052	0.067
C (13)	-0.022	-0.02	-0.024	N (13)	0.041	0.039	0.043	N (13)	0.028	0.021	0.034	N (13)	0.025	0.023	0.027
C (14)	-0.021	-0.02	-0.021	C (14)	-0.023	-0.022	-0.023	C (14)	-0.021	-0.02	-0.021	C (14)	-0.025	-0.025	-0.026
H (15)	0.069	0.07	0.069	H (15)	0.035	0.037	0.033	H (15)	0.046	0.047	0.044	H (15)	0.049	0.05	0.047
H (16)	0.063	0.064	0.062	H (16)	-0.018	-0.015	-0.022	H (16)	0.036	0.038	0.033	H (16)	0.033	0.035	0.032
H (17)	0.062	0.062	0.063	H (17)	0.038	0.04	0.035	H (17)	-0.005	-0.001	-0.009	H (17)	0.034	0.036	0.032
H (18)	0.069	0.069	0.069	H (18)	0.045	0.047	0.044	H (18)	0.038	0.04	0.036	H (18)	0.048	0.05	0.047
H (19)	0.08	0.08	0.079	H (19)	0.045	0.047	0.043	H (19)	0.045	0.046	0.043	H (19)	0.054	0.056	0.052
H (20)	0.078	0.078	0.077	H (20)	0.038	0.038	0.039	H (20)	0.09	0.095	0.085	H (20)	0.062	0.063	0.06
H (21)	0.08	0.081	0.08	H (21)	0.065	0.068	0.062	H (21)	0.042	0.041	0.044	H (21)	0.059	0.06	0.058
H (22)	0.034	0.034	0.035	H (22)	0.078	0.076	0.08	H (22)	0.074	0.073	0.074	H (22)	0.068	0.066	0.069
H (23)	0.035	0.035	0.035	H (23)	0.08	0.079	0.082	H (23)	0.091	0.09	0.093	H (23)	0.069	0.069	0.069
H (24)	0.047	0.046	0.047	H (24)	0.096	0.095	0.098	H (24)	0.077	0.075	0.079	H (24)	0.07	0.069	0.071
H (25)	0.022	0.022	0.022	H (25)	0.078	0.077	0.079	H (25)	0.071	0.068	0.075	H (25)	0.069	0.068	0.07
H (26)	0.022	0.022	0.022	H (26)	0.046	0.045	0.046	H (26)	0.051	0.051	0.05	H (26)	0.059	0.065	0.053
H (27)	0.038	0.038	0.038	H (27)	0.051	0.049	0.053	H (27)	0.042	0.041	0.043	H (27)	0.058	0.052	0.063
H (28)	0.038	0.038	0.038	H (28)	0.06	0.059	0.06	H (28)	0.051	0.049	0.053	H (28)	0.046	0.043	0.048
H (29)	0.021	0.021	0.021	-	-	-	-	-	-	-	-	-	-	-	-
H (30)	0.005	0.003	0.007	-	-	-	-	-	-	-	-	-	-	-	-
H (31)	0.032	0.031	0.032	-	-	-	-	-	-	-	-	-	-	-	-
H (32)	-0.001	0	-0.001	-	-	-	-	-	-	-	-	-	-	-	-
H (33)	0.061	0.061	0.062	-	-	-	-	-	-	-	-	-	-	-	-
H (34)	0.036	0.036	0.036	-	-	-	-	-	-	-	-	-	-	-	-
Where	F0: Mulliken atomic charges				f+: Nucleophilic attack			f-: Electrophilic attack							

**Table 10:** Comprehensive comparison of ILs as corrosion inhibitors at high concentration.

Inhibitor/concentration	Medium	Metal	(EIS) IE %	Refs.
IL_I_(100ppm)	1 M HCl	Carbon Steel	96.69	Present Study
IL_II_(100ppm)	1 M HCl	Carbon Steel	96.47	
IL_III_(100ppm)	1 M HCl	Carbon Steel	97.21	
IL_IV_(100ppm)	1 M HCl	Carbon Steel	96.28	
*1-butyl-3-methylimidazolium methane sulfonate (800 ppm)*	M H_2_SO_4_	Carbon Steel	91.1	58
*-hexadecyl-3-(4-methylbenzyl)-1 H-imidazol-3-ium chloride(120 ppm)*	1 M HCl	Carbon Steel	92.90	59
1-hexadecyl-3-(4-nitrobenzyl)-1 H-imidazol-3-ium chloride*(120 ppm)*	1 M HCl	Carbon Steel	94.50	59

## Conclusion

The structural characterization of the synthesized ionic liquids (IL_I_–IL_IV_) was comprehensively established using elemental analysis, FT-IR spectroscopy, and ^1^H-NMR spectroscopy. The elemental analysis results confirmed that the experimental composition of all inhibitors closely matched their theoretical values. FT-IR spectroscopy validated the presence of key functional groups, while ^1^H-NMR spectra provided deeper insights into the molecular environment and proton interactions within each compound.

Various electrochemical approaches, including PP, EIS, and EFM, showed that all four inhibitors effectively reduced the corrosion of C-steel by 1 M HCl, reaching up to 97% protection at an optimal concentration of 100 ppm. These inhibitors mainly function as dual-action inhibitors, where their adsorption onto the metal surface plays a crucial role in lowering the C.R. Further quantum chemical calculations confirmed these findings, revealing that IL_II_–IL_IV_ exhibited better performance due to their ability to coordinately share electrons more effectively, which is attributed to their aromatic ring structures and narrower energy gaps.

Overall, both experimental and theoretical results confirm that the investigated ionic liquids serve as effective corrosion inhibitors for C-steel in acidic solutions.

## Supplementary Information

Below is the link to the electronic supplementary material.

Supplementary Material 1

## Data Availability

The datasets used and/or analysed during the current study available from the corresponding author on reasonable request.
