# Supplementary material for: Synthesis and assessment of ionic liquid derived from benzalkonium chloride as corrosion inhibitor for carbon steel
Source: Sci Rep. 2026 Feb 9;16:5361. doi: 10.1038/s41598-025-14549-0 (PMC12887016; doi:10.1038/s41598-025-14549-0)
Supplement: Supplementary file 1 — Supplementary Material 1 [file 41598_2025_14549_MOESM1_ESM.docx]

**Supplementary file**

**Synthesis and Assessment of Ionic Liquid Derived from Benzalkonium Chloride as Corrosion Inhibitor for Carbon Steel**

Ashraf M. Ashmawy^1^, Reda Abdel-Hameed ^1^, Odeh A.O. Alshammari^2^, Maher I. Nessim*^3^, Modather F. Hussein ^4^_,_ Abdalrahman G. Al-Gamal^3^

^1^Chemistry Department, Faculty of Science (boys), Al-Azhar University, 11884, Egypt.

^2^Department of Chemistry, College of Science, University of Ha’il, 81442, Hail, Saudi Arabia.

^3^Egyptian Petroleum Research Institute (EPRI), Nasr City 11727, Cairo, Egypt.

^4^Chemistry Department, Faculty of Science, Al-Azhar University, Asyut Branch, Assiut 71524, Egypt.

***** Corresponding authors: Maher I. Nessim(maherni@yahoo.com)


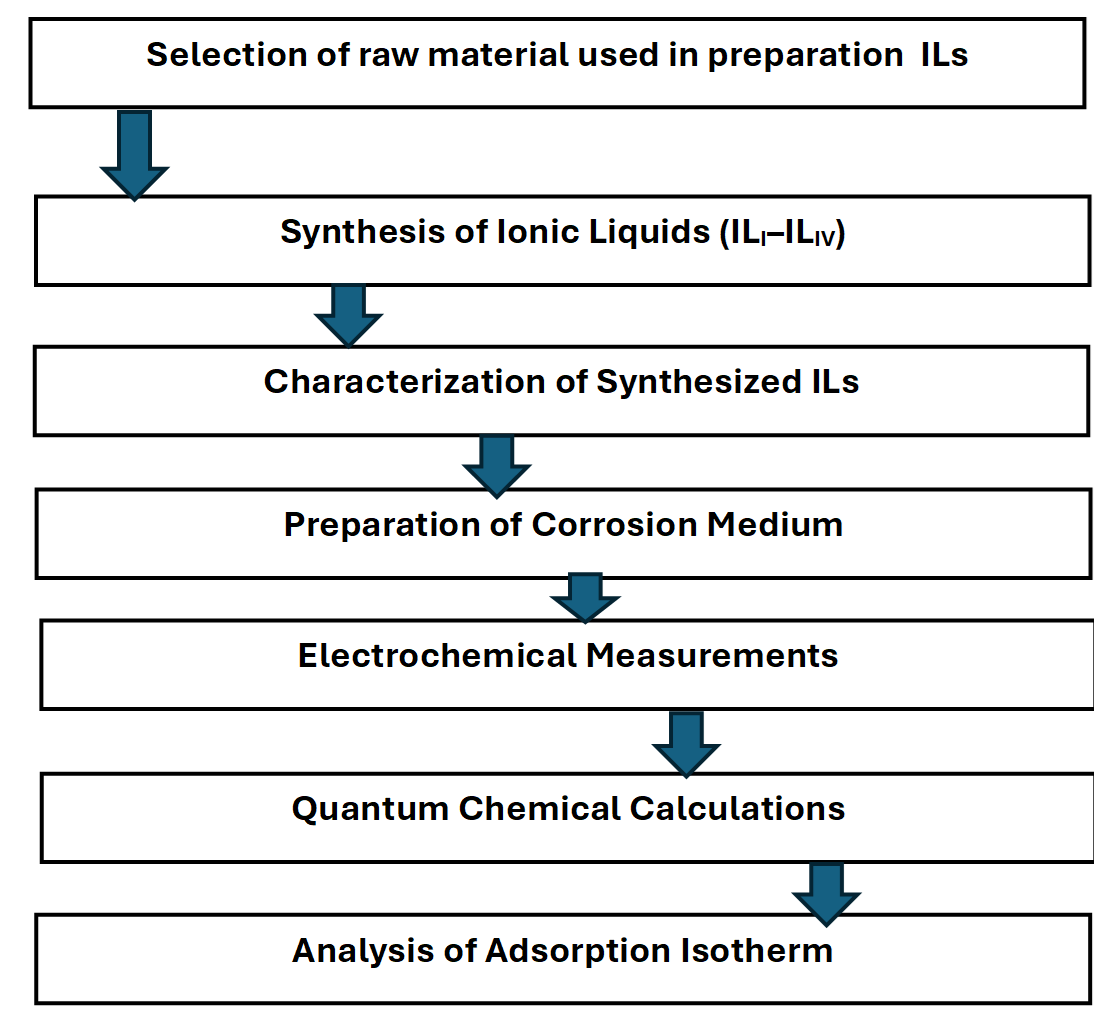


Fig. S1 Flowchart of the research methodology for synthesis, characterization, and corrosion inhibition assessment of ILs


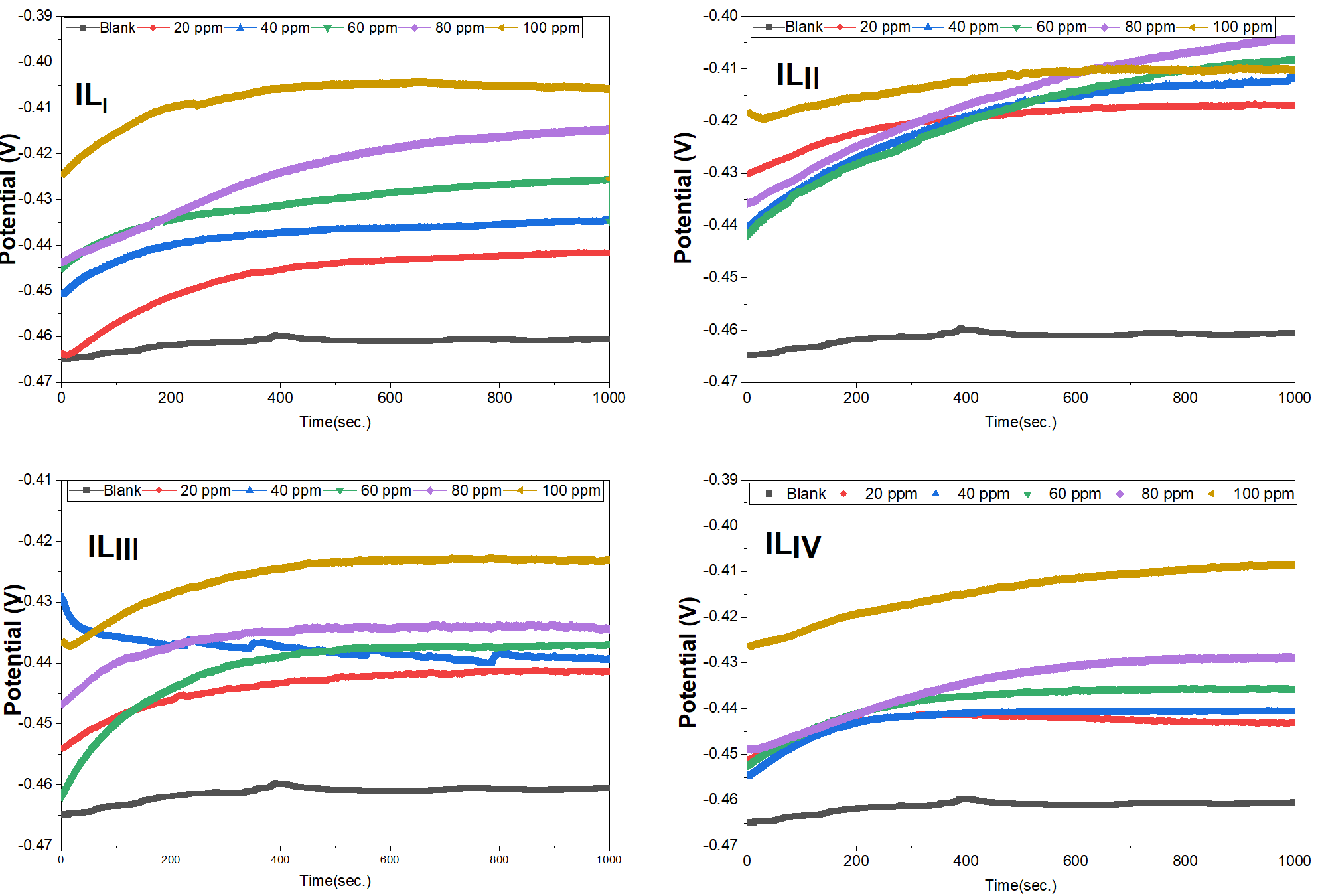


Fig. S2 Plot of OCP against time for ILs.

**
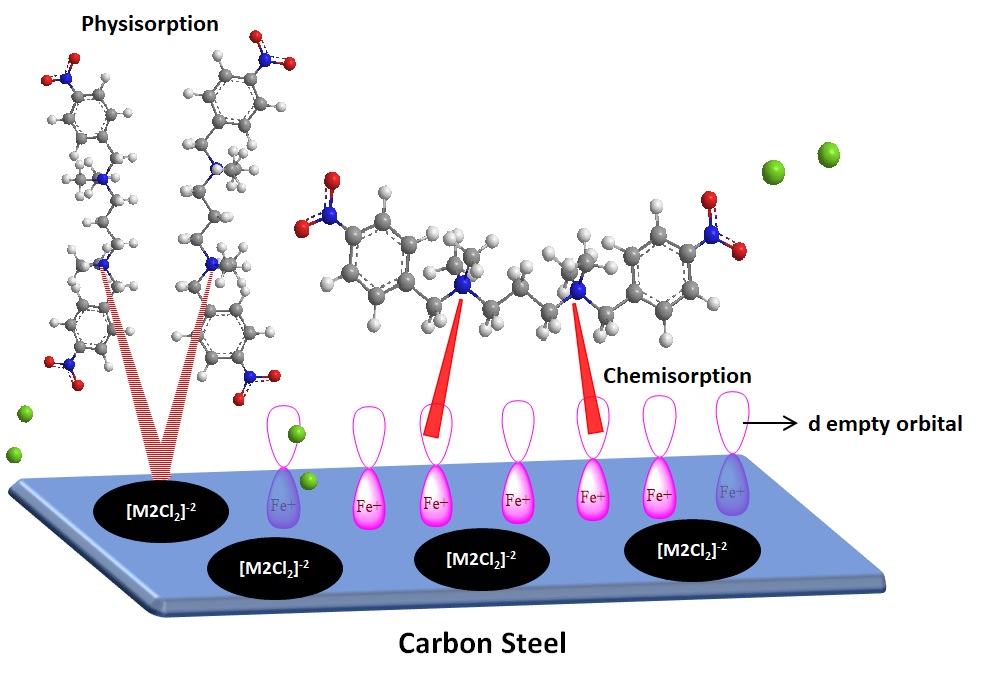
**

Fig. S3: illustrate the Mechanism of Inhibition for prepared ILs compounds.
